# Supplementary material for: Effect of Different Phosphates on Pyrolysis Temperature-Dependent Carbon Sequestration and Phosphorus Release Performance in Biochar
Source: Molecules. 2023 May 8;28(9):3950. doi: 10.3390/molecules28093950 (PMC10180186; doi:10.3390/molecules28093950)
Supplement: Supplementary file 1 [file molecules-28-03950-s001.zip › molecules-2342687-supplementary.pdf]

Supporting Information

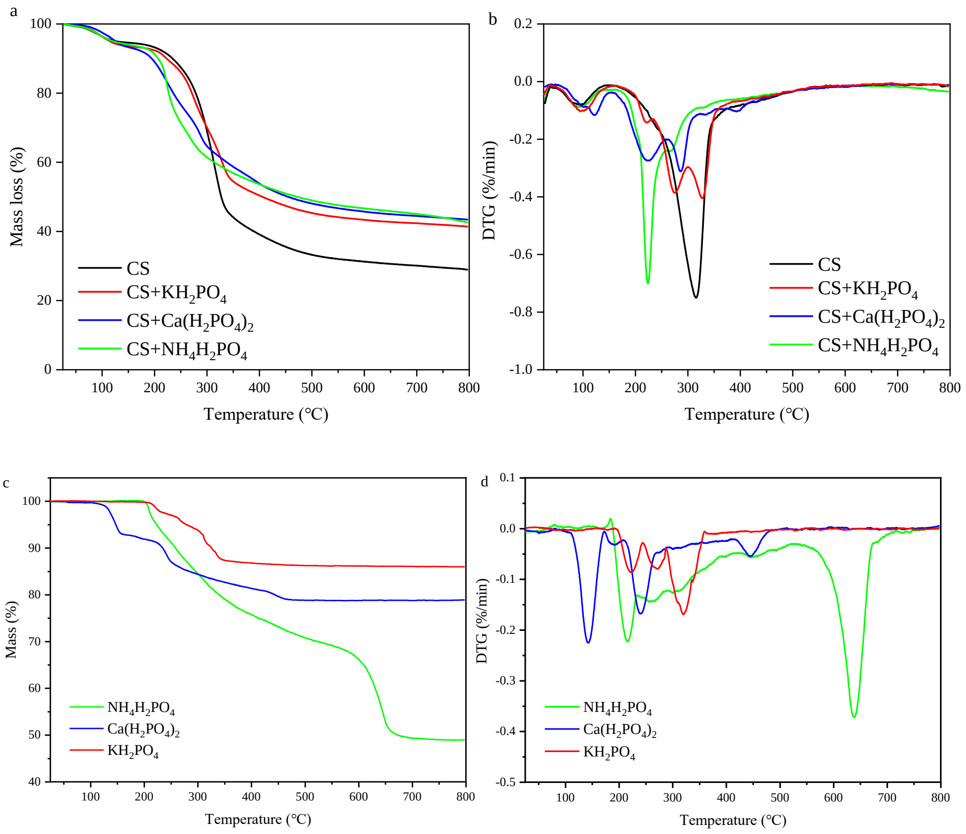

Figure S1 TG and DTG curves of corn straw with or without phosphate (a, b) and single phosphate (c, d) at a heating rate of 10 °C min^{-1}

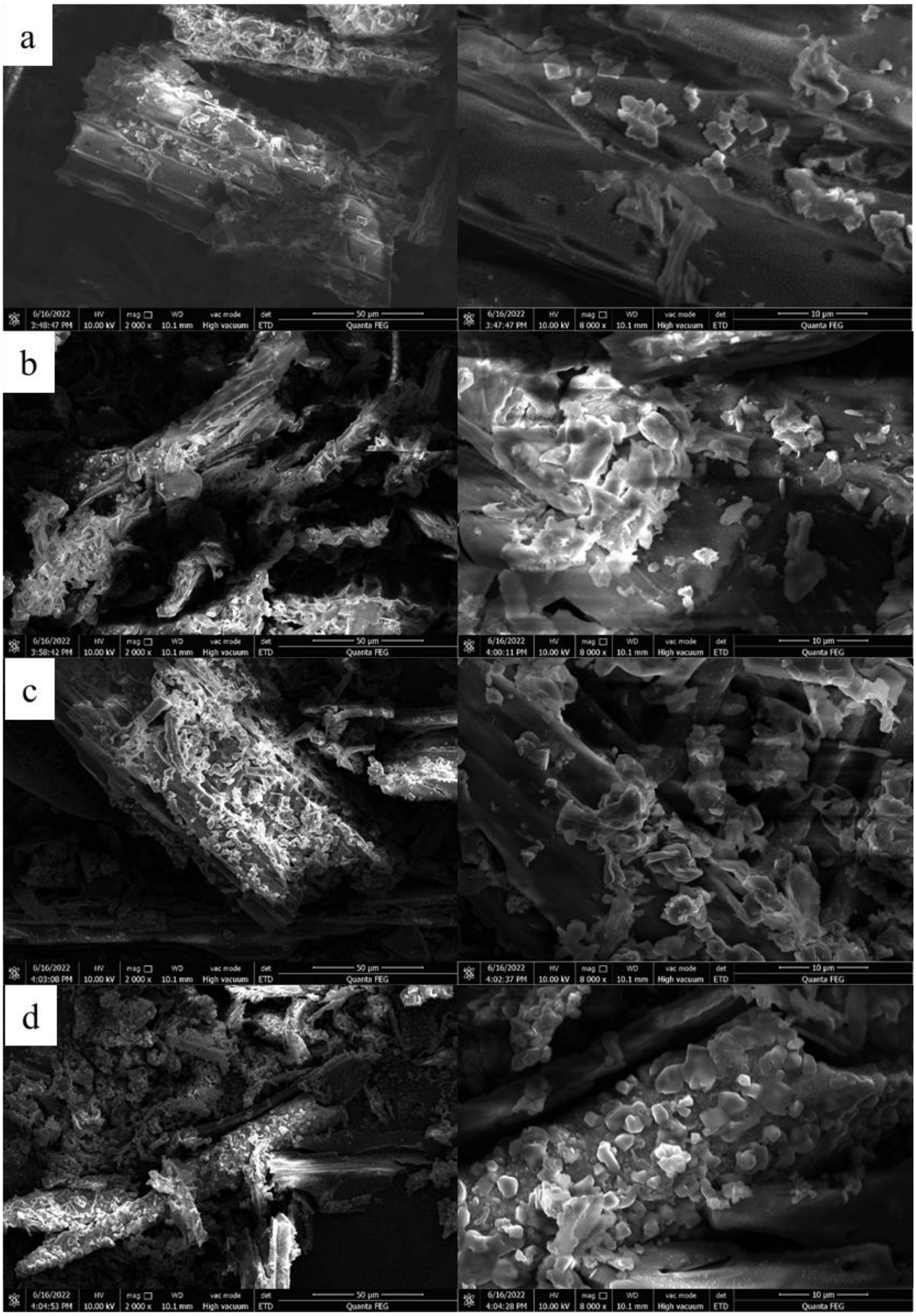
a
b
c
d
50 μm
10 μm

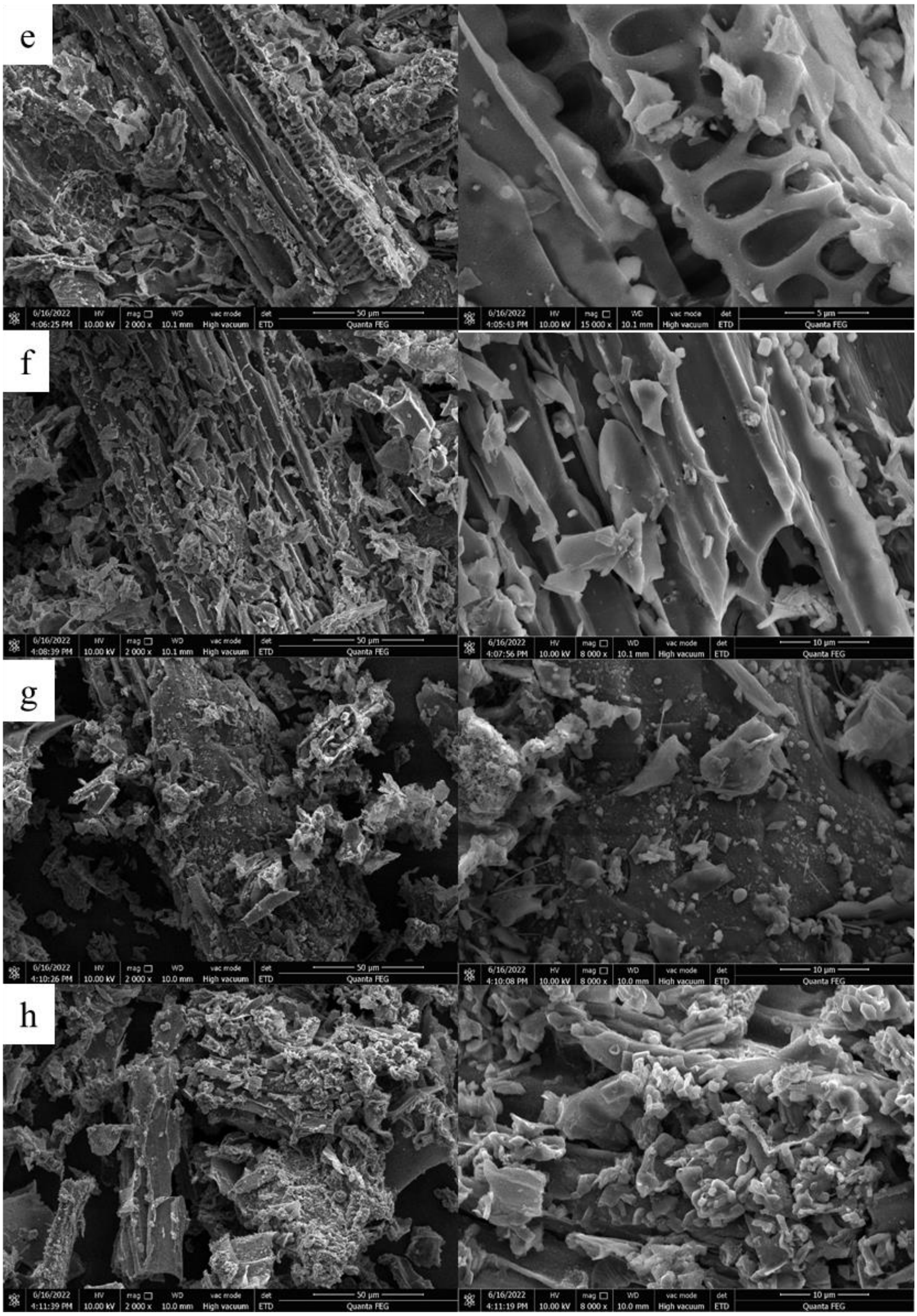

e
6/16/2022 HV mag WD vac mode det 50 μm
4:06:25 PM 10.00 kV 2 000 x 10.1 mm High vacuum ETD Quanta FEG
6/16/2022 HV mag WD vac mode det 5 μm
4:05:43 PM 10.00 kV 15 000 x 10.1 mm High vacuum ETD Quanta FEG
f
6/16/2022 HV mag WD vac mode det 50 μm
4:08:39 PM 10.00 kV 2 000 x 10.1 mm High vacuum ETD Quanta FEG
6/16/2022 HV mag WD vac mode det 10 μm
4:07:56 PM 10.00 kV 8 000 x 10.1 mm High vacuum ETD Quanta FEG
g
6/16/2022 HV mag WD vac mode det 50 μm
4:10:26 PM 10.00 kV 2 000 x 10.0 mm High vacuum ETD Quanta FEG
6/16/2022 HV mag WD vac mode det 10 μm
4:10:08 PM 10.00 kV 8 000 x 10.0 mm High vacuum ETD Quanta FEG
h
6/16/2022 HV mag WD vac mode det 50 μm
4:11:39 PM 10.00 kV 2 000 x 10.0 mm High vacuum ETD Quanta FEG
6/16/2022 HV mag WD vac mode det 10 μm
4:11:19 PM 10.00 kV 8 000 x 10.0 mm High vacuum ETD Quanta FEG

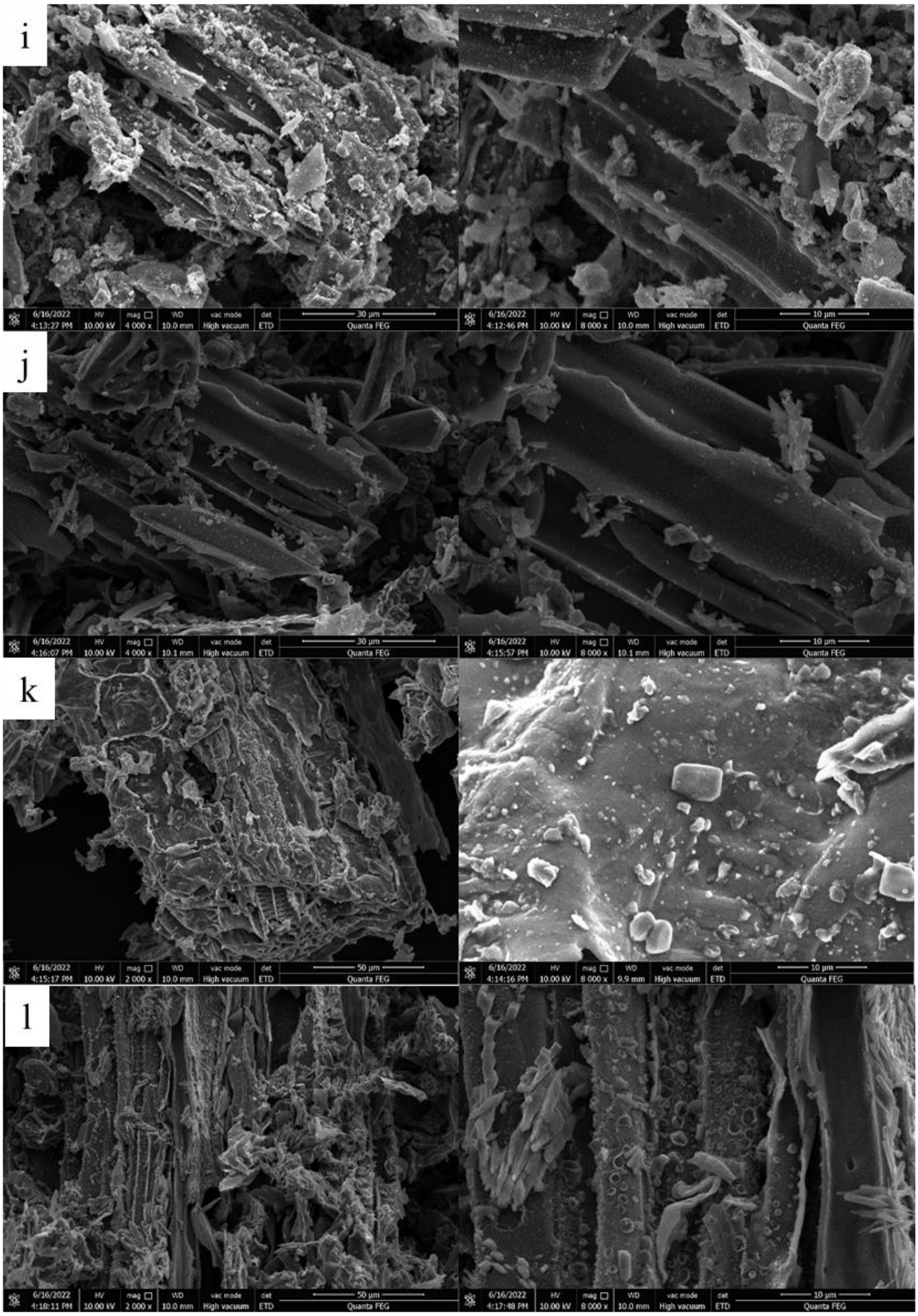

Figure S2 SEM image of the biochars (a-CSB300, b-CSB300+K, c-CSB300+Ca, d-CSB300+N, e-CSB500, f-CSB500+K, g-CSB500+Ca, h-CSB500+N, i-CSB700, j-CSB700+K, k-CSB700+Ca, l-CSB700+N)

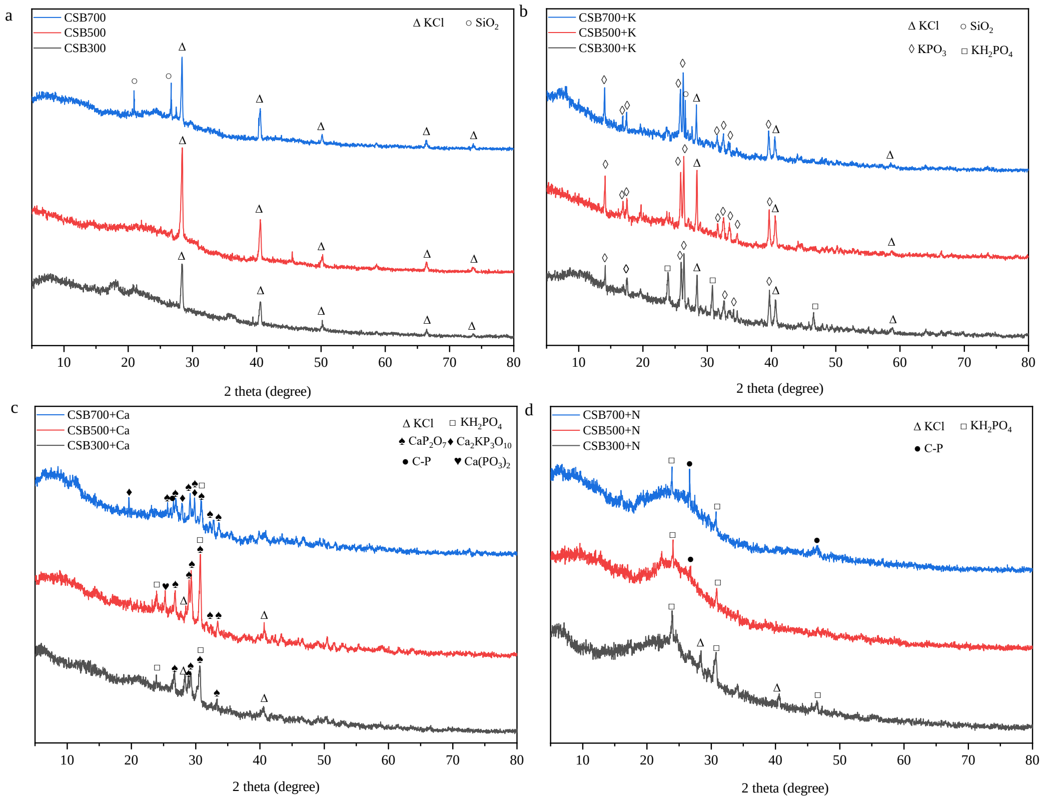

Figere S3 XRD patterns of the biochars

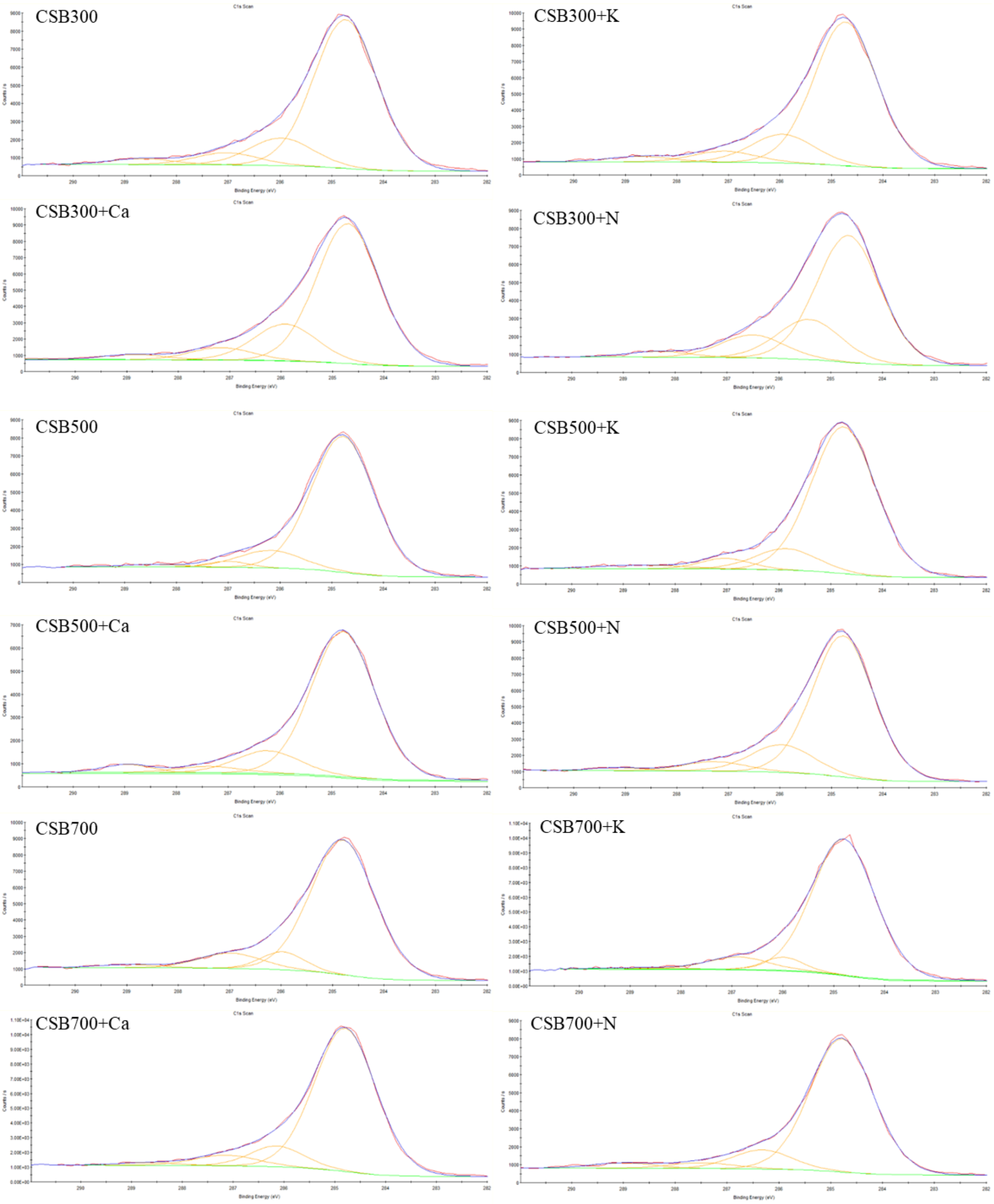

Figure S4 XPS spectra of C_{1s} in biochar

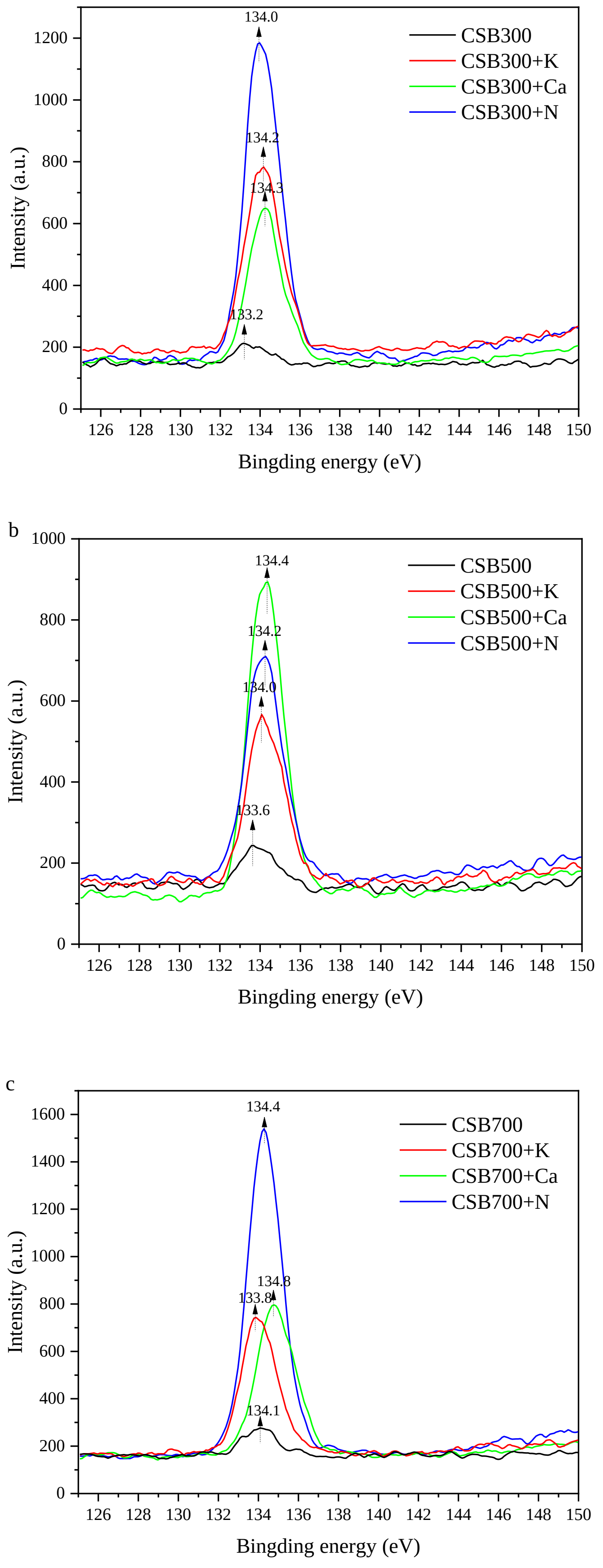

Figure S5 XPS spectra of P_{2p} in biochar

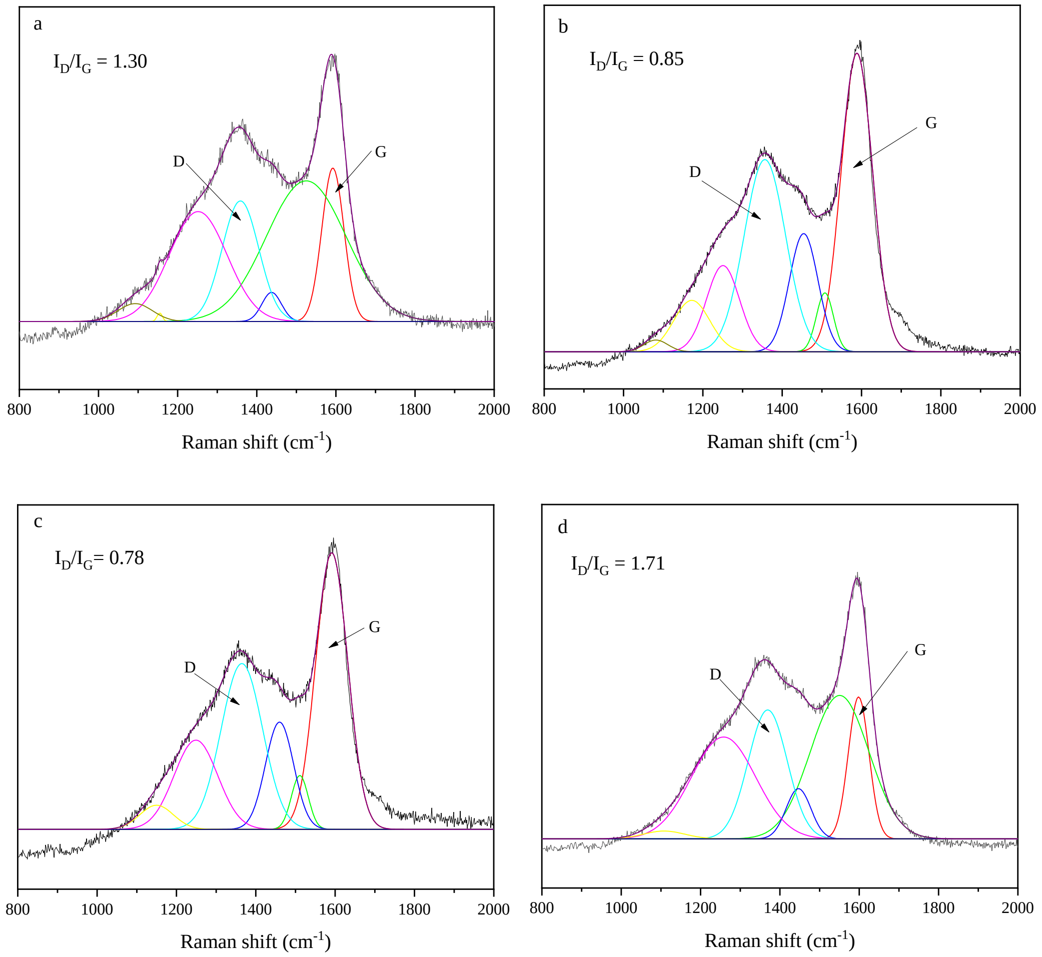

Figure S6 Raman spectra of biochars (a-CSB500, b-CSB500+K, c-CSB500+Ca, d-CSB500+N)

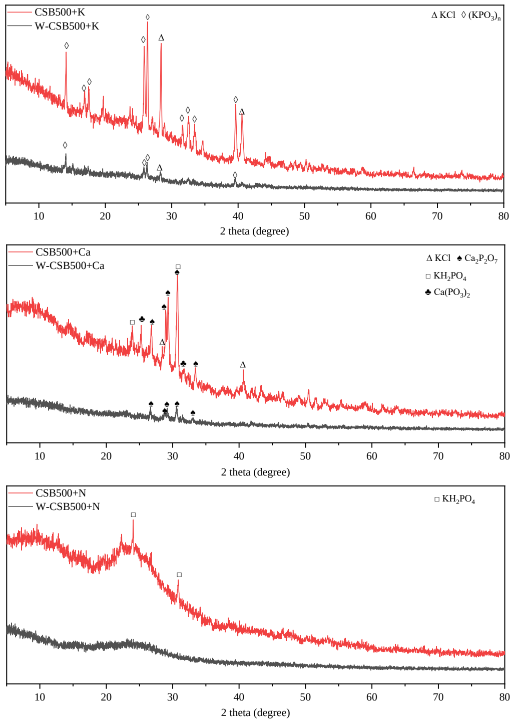

Figure S7 XRD of biochar pyrolyzed at 500 °C before and after P release

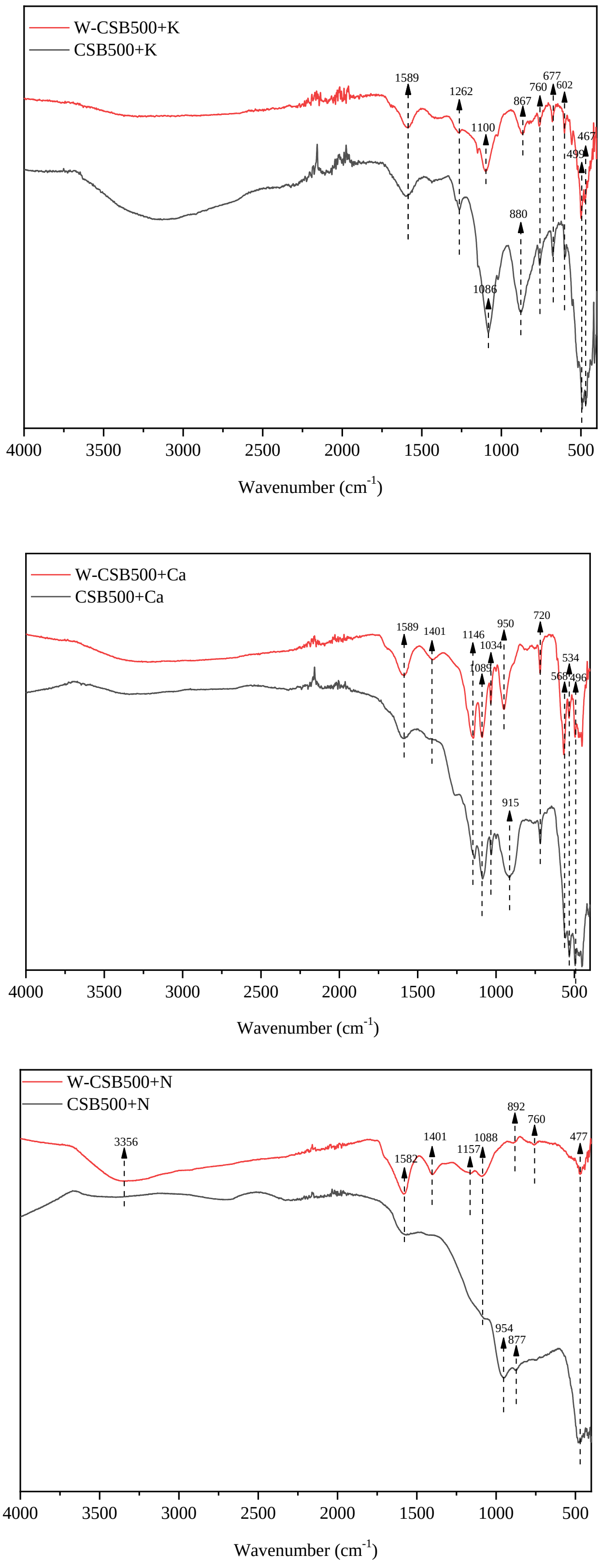

Figure S8 FTIR spectra of biochar pyrolyzed at 500 °C before and after P release

Table S1 Porous structural parameters of biochar pyrolyzed at 500℃

	BET	Total pore volume (cm^3/g)	Micropore volume (cm^3/g)	Percentage of micropore volume (%)	Average pore width (nm)
CSB500	9.85	0.014	0.0030	21.43	6.17
CSB500+K	37.65	0.046	0.0079	17.17	4.87
CSB500+Ca	9.95	0.020	0.0033	16.50	7.59
CSB500+N	34.46	0.037	0.0060	16.22	4.60

The specific surface area (SSA), micropore volume was calculated by the Brunauer, Emett and Teller (BET) equation and the Horvath-Kawazoe equation.
